# LncRNA FLVCR1-AS1 mediates miR-23a-5p/SLC7A11 axis to promote malignant behavior of cervical cancer cells

**DOI:** 10.1080/21655979.2022.2059958

**Published:** 2022-04-23

**Authors:** Xi Zhou, Xia Zhao, ZhouYi Wu, Yan Ma, Heng Li

**Affiliations:** a Department of Gynecology, The First Affiliated Hospital of University of South China Hengyang, Hengyang City, Hunan Province, China; b Medical School, Hunan University of Chinese Medicine, Changsha City, Hunan Province, China; c Department of Gynecology, Loudi Central Hospital, Loudi City, Hunan Province, China

**Keywords:** Cervical cancer, HeLa cells, FLVCR1-AS1, MicroRNA-23a-5p, solute carrier family 7 member 11

## Abstract

Cervical cancer (CC) is the most common gynecological malignant tumor in the world. Long non-coding RNA (lncRNAs) plays an important role in cell activities of various cancers including CC. This study aims to reveal the biological function of FLVCR1-AS1 in CC and clarify its possible mechanism of action. The findings suggest that the expression of FLVCR1-AS1 was elevated in CC tissues and cell lines, and that high expression of FLVCR1-AS1 was associated with poor prognosis of CC patients. In addition, knockdown of FLVCR1-AS1 could inhibit the proliferation and migration, invasion and epithelial–mesenchymal transformation (EMT) of CC cells, as well as accelerating apoptosis, to inhibit the development of CC. In addition, via the dual-luciferase reporting assay and RIP assay were confirmed that FLVCR1-AS1 acted as a competitive endogenous RNA to inhibit the expression of microRNA (miR)-23a-5p, and miR-23a-5p targeted the 3’-untranslated region site of Solute carrier family 7 member 11 (SLC7A11) and negatively regulated the expression of SLC7A11. Functional rescue experiments showed that miR-23a-5p inhibitors reversed the inhibitory effect of FLVCR1-AS1-silencing on proliferation, EMT, migration and invasion, and the promoting impact of apoptosis of CC cells. In addition, SLC7A11 rescued the effect of miR-23a-5p overexpression on progression of CC cells. In conclusion, FLVCR1-AS1 is involved in the malignant phenotype of CC cells through miR-23a-5p/SLC7A11 axis, which may provide a beneficial direction for the treatment of CC.

## GRAPHICAL ABSTRACT

**Figure:**
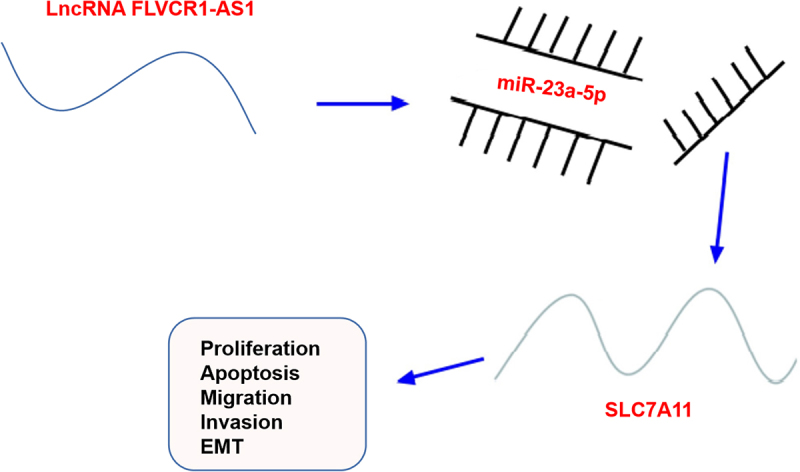

## Highlights

1. FLVCR1-AS1 and SLC7A11 are elevated, but miR-23a-5p is declined in CC tissues and cells;

2. Down-regulation of FLVCR1-AS1 can inhibit the proliferation, migration, invasion, and EMT of CC cells;

3. Down-regulation of miR-23a-5p could reverse the inhibitory effect of down-regulation of FLVCR1-AS1 on proliferation, migration, invasion, and EMT of CC cells;

4. LncRNA FLVCR1-AS1 influences the biological functions of CC cells via miR-23a-5p to target SLC7A11.

## 1. Introduction

Cervical cancer (CC) is the crucial cause of cancer-associated deaths in women aged 20–39 all over the world [1]. Presently, the critical clinical treatment strategies for CC cover surgery, chemotherapy, and radiotherapy. Nevertheless, radiotherapy and chemotherapy are available to kill a considerable number of normal cells owing to the limitation of the adaptation stage of surgical cure [2]. Nevertheless, these traditional therapy methods are unable to effectively cure CC, and infiltration, metastasis, and recurrence remain the crucial reason of death [3]. As the development of molecular biology and genomics, molecular targeted treatment has achieved breakthrough in the cure of cancer [4]. Studies have manifested molecular targeted therapy distinctly influences the cell advancement with recurrence in CC with few side effects [5]. Consequently, it is imperative to seek for new targeted cure targets and prognostic biomarkers to strengthen the survival rate in CC patients.

Long non-coding RNA (lncRNA), a group of non-coding RNA with over 200 nucleotides, implicates in numerous physiological and pathological processes of malignant tumors [6]. Numerous evidences elaborate lncRNA is the crucial regulator of CC progression. For instance, lncRNA FBXL19-AS1, as the sponge of microRNA (miRNA, miR)-193a-5p, boosts the advancement of CC via elevating COL1A1 [2]. LncRNA LIPE-AS1 forecasts the declined survival rate of CC and facilitates its progression via modulating the miR-195-5p/MAPK pathway [7]. The evidence has illuminated FLVCR1-AS1 belonging to lncRNA performs as the tumor promoter in certain cancers. For instance, FLVCR1-AS1 boosts osteosarcoma cell advancement via activating the wnt/β-catenin pathway [8]. LncRNA FLVCR1-AS1 boosts the progression with epithelial–mesenchymal transition (EMT) of ovarian cancer cells via mediating the miR-513/YAP1 signaling pathway [9]. Nevertheless, the regulatory mechanism and biological functions of FLVCR1-AS1 in CC are still uncertain.

The extremely crucial molecular mechanism in which mediates the cascade of cancer cell metastasis is EMT [10]. EMT is characterized via the loss of epithelial features and the acquisition of mesenchymal function, which further endow cancer cell metastasis with stemness capabilities [11]. As reported, numerous lncRNAs impact CC advancement via modulating the process of EMT. For instance, the LINC00861/miR-513b-5p axis restrains the process of EMT in CC cells via the PTEN/AKT/the mammalian target of rapamycin (mTOR) pathway, thereby influencing CC progression [12]. Lnc-RNASNHG3 boosts SiHa cell advancement via activating the EMT pathway [13]. Nevertheless, the latent action of FLVCR1-AS1 in CC concerning the progress of EMT remains uncertain.

Therefore, this experiment is designed to explore the biological function and action mechanism of FLVCR1-AS1 in CC. First, the expression level of FLVCR1-AS1 was detected in CC tissues and cells compared with normal tissues and cells, and the correlation of the clinicopathology, prognosis of CC patients, and the expression of FLVCR1-AS1 was analyzed. Secondly, CCK-8 assay, colony formation assay, Transwell assay, flow cytometry determination and related molecular protein assay were performed to determine the effect of FLVCR1-AS1 on proliferation migration, invasion, EMT, and apoptosis of CC cells. Finally, the downstream miRNA and target genes were confirmed and identified by dual-luciferase reporting assay and RIP assay. The results explained why FLVCR1-AS1 can promote the malignant behavior of CC cells.

## 2. Materials and methods

### 2.1. Clinical specimens

From March 2018 to October 2020, collection of 40 CC tumor tissues, adjacent normal tissues, and serum samples was from 40 CC patients via surgery in The First Affiliated Hospital of University of South China Hengyang. Histopathological and clinical diagnosis of the tissue specimens was via the pathologist and storing was conducted. Authorization of the research was via the Ethics Committee of The First Affiliated Hospital of University of South China Hengyang, and obtainment of all participants’ written informed consent was conducted (Approval number 201713772).

### 2.2. Cell culture and transfection

Human healthy cervical cells (HUCEC) and human CC cells (HeLa, Caski, C-33A, AV3) were applied (all American Type Culture Library, Rockville, MD, USA). Culture of cells was in Roswell Park Memorial Institute (RPMI) 1640 (Gibco, NY, USA) medium covering 10% fetal bovine serum (FBS) (Gibco, NY, USA) and 1% penicillin/streptomycin (Gibco, NY, USA). Selection of Hela cells is as the auxiliary research object owing to its extremely elevated difference [2].

Division of HeLa cells was into nine groups: The miR/sh-negative control (NC), the sh-FLVCR1-AS1#1/#2, the miR-23a-5p, the sh-FLVCR1-AS1#1 + in-NC/in-miR-23a-5p and the miR-23a-5p + Oe-NC/- Solute carrier family 7 member 11 (SLC7A11). The sh-FLVCR1-AS1#1#2 and its sh-NC with SLC7A11 and Oe-NC were employed (all Shanghai GenePharma, China). Construction of miR-23a-5p mimic/inhibitor and NC mimic/inhibitor was performed from GenePharma. Transfection of HeLa cells was via all oligonucleotides (50 nM) and vectors (4.0 μg) adopting Lipofectamine 3000 (Invitrogen) [14].

### 2.3. Subcellular localization analysis

Separation of the cytoplasmic and nuclear RNA of HeLa cells was via adopting the PARIS Kit (Ambion, Austin, TX, United States). Then, detection of lncRNA FLVCR1-AS1 in the cytoplasmic and nuclear RNA of HeLa cells was via adopting reverse transcription quantitative polymerase-chain reaction (RT-qPCR). Adoption of U6 and glyceraldehyde-3-phosphate dehydrogenase (GAPDH) was as nuclear and cytoplasmic control [15].

### 2.4. RT-qPCR

Extraction of total RNA from CC tumor tissue and HeLa cells was via adopting Trizol reagent (Invitrogen, USA). Reverse transcription of lncRNA/mRNA and miRNA was performed adopting lnRcute lncRNA a complementary DNA (cDNA) kit (TIANGEN, China) and Rcute Plus miRNA cDNA kit, separately (TIANGEN, China). Test was via exerting SYBR Green kit (Thermo Fisher Scientific, Waltham, MA, USA) and Mx3005P QPCR system (Agilent Technologies, Santa Clara, CA, USA). Adoption of U6 and GAPDH was as loading controls for miRNA and mRNA/lncRNA, separately. The primer sequence was manifested in Table 1 [16].

**Table 1.** Primer sequences

Genes	Primer sequences (5’-3’)
FLVCR1-AS1	F: 5’-GTCTCTCCCTCC-3’
	R: 5’-CCGTCTCGGTTTTC-3’
MiR-23a-5p	F: 5’-GGGGUUCCUGGGGAUGGGAUUU-3’
	R: 5’-GTGCAGGGTCCGAGGT-3’
SLC7A11	F: 5’-CTCGCTTCGGCAGCACA-3’
	R: 5’-GGCGTCTTTAAAGTTCTGCG-3’
N-cadherin	F: 5’-CCATCAAGCCTGTGGGAATC-3’
	R: 5’-GCCGCTTTAAGGCCCTCAT-3’
Snail	F: 5’-TCCAGCAGCCCTACGACCAG-3’
	R: 5’-AGGCCGAGGTGGACGAGAA-3’
Vimentin	F: 5’-TCTGGATTCACTCCCTCTGGTT-3’
	R: 5’-ATCGTGATGCTGAGAAGTTTCGT-3’
E-cadherin	F: 5’-CGAGAGCTACACGTTCACGG-3’
	R: 5’-GGGTGTCGAGGGAAAAATAGG-3’
U6	F: 5’-CTCGCTTCGGCAGCACA-3’
	R: 5’-AACGCTTCACGAATTTGCGT-3’
GAPDH	F: 5’-TCCCATCACCATCTTCCA-3’
	R: 5’-CATCACGCCACAGTTTTCC-3’

F, forward; R, reverse.

### 2.5. Western blot for detection of EMT pathway-linked proteins

After collecting and treating HUCEC and HeLa cells, extraction of total protein in the cells was conducted, and examination of the protein concentration was via adopting bicinchoninic acid. Separation of 35 μg protein was via 10% sulfate polyacrylamide gel electro-pheresis, and electroblot of the separated protein was onto polyvinylidene fluoride membrane with constant current (210 mA); After blocking of 5% bovine serum albumin, addition of the primary antibodies at dilution of 1: 1000 and incubation were conducted (E-cadherin/N-cadherin/Vimentin/Snail/GAPDH, all Abcam). Rinse of free antibodies was via tris-buffered saline with Tween 20 (TBST), and separate accretion of enzyme-labeled goat anti-rabbit immunoglobulin G (IgG) (1: 50,000) or enzyme-labeled goat anti-mouse IgG (dilution of 1: 50,000) and incubation were performed; 1 × TBST membrane 10 min × 3, horseradish peroxidase chemiluminescence color development [13].

### 2.6. Examination of cell counting kit 8 (CCK8)

Separate seeding of the logarithm 1 × 10^3^ HeLa cells was in 96-well plates (1 × 10^3^ cells/well). Addition of 10 μL CCK8 reagent (Dojindo, Kumamoto, Japan) was to each well at the designated time points (0, 24, 48, and 72 h). After 2 h, record of the optical density was at 450 nm adopting the microplate reader [7].

### 2.7. Colony formation experiment

Culture of the logarithmic cells of 1 × 10^3^ was in 6-well plates. After fixation in methanol (Solarbio, Beijing, China), treatment of the colonies was with crystal violet (Sigma-Aldrich). Manual counting of visible colonies was performed under a microscope (Olympus, Tokyo, Japan) [17].

### 2.8. Flow cytometry

Test of cell apoptosis was via exerting Annexin V Kit (Beyotime, Beijing, China). Detachment of the cells was with 0.25% trypsin, removal of digestive juice was conducted, and placing of the cells was in the previously collected medium. Centrifugation at 12,000 RPM and abandonment of the supernatant were performed. Collection of the cell precipitates and production of the cell suspension with phosphate buffer saline were conducted. Centrifugation again and removal of the supernatant were conducted; Addition of Annexin V fluorescein-isothiocyanate and 300 mL propidium iodide labeling solution and incubation were performed [7].

### 2.9. Transwell migration and invasion analysis

Assessment of cell migration and invasion capabilities was conducted. Transfection of 2 × 10^4^/200 μL cells was with 8-μm well filter (Millipore, Germany); After plasmid transfection of 48 h, coating or uncoating of 50 μL Matrigel (BD Biosciences) was in serum-free medium, and addition of Dulbecco’s Modified Eagle Medium covering 10% FBS was as attractant. Migration and invasion were performed after incubation: removal of the cells without migrating or invading was conducted, and fixation of the cells migrating to the bottom of the membrane was with 4% paraformaldehyde. Staining was with crystal violet solution, and observation was conducted under the 100 × microscope. Counting of all cells is performed under five randomly selected microscopes [18].

### 2.10. Glutathione (GSH)/glutathione disulfide (GSSG) method

Detection of reduced GSSG and GSH in HeLa cells was via adopting the glutathione detection kit. Carrying out of all operations was in the light of the manufacturer’s instructions. Design and performance of all experiments were three times [19].

### 2.11. The luciferase activity assay

Co-transfection of pGL3-basic vector (GenePharma, Shanghai, China) with FLVCR1-AS1- wild type (WT)/mutant type (MUT) and miR-23a-5p mimic or mimic-NC was into HeLa cells adopting lipofectamine 2000 reagent ThermoFisher). Correspondingly, Co-transfection of the pGL3-basic vector of SLC7A11-WT or SLC7A11-MUT and miR-23a-5p mimic or mimic NC was into HeLa cells exerting Lipofectamine 2000 reagent. Examination of luciferase activity is via adopting the luciferase detection system after 48 h [20].

### 2.12. RNA immunoprecipitation (RIP) assay

After collection of HeLa and HCC94 cells, analysis of the transfected cells was conducted adopting Magna RIPTM RNA-binding protein immunoprecipitation kit (Millipore, Bedford, Massachusetts, USA). Then, incubation of the cells is with anti-ago2 antibody (Millipore) or negative control IgG (Millipore), and detection of the enrichment of FLVCR1-AS1, miR-23a-5p, and SLC7A11 in the cell lysate is performed [7].

### 2.13. Statistical analysis

Analysis of the data was via adopting SPSS 21.0 (SPSS, Inc, Chicago, IL, USA) statistical software. Through Kolmogorov–Smirnov test, the data were presented in normal distribution. Manifestation of the measurement data was as mean ± standard deviation. The two-group comparison was via adopting t test; The comparison among the multiple groups was via adopting one-way analysis of variance (ANOVA), and the pairwise comparison after ANOVA analysis was via exerting Fisher’s least significant difference t test (LSD-t). Manifestation of enumeration data was in rate or percentage, and comparative analysis was via adopting the chi-square test. *P* < 0.05 was accepted as indicative of remarkable differences.

## 3. Results

FLVCR1-AS1 and SLC7A11 are highly expressed, while miR-23a-5p are lowly expressed in CC patients. Down-regulation of FLVCR1-AS1 inhibits HeLa cell proliferation, migration, invasion, and EMT, but promotes cell apoptosis. FLVCR1-AS1 acts as a sponge for miR-23a-5p, and miR-23a-5p targets SLC7A11. FLVCR1-AS1 affects the malignant behavior of HeLa cells through the regulatory axis of FLVCR1-AS1/miR-23a-5p/SLC7A11.

### 3.1. Overexpression of FLVCR1-AS1 in CC tissues and cell lines predicts unpleasing prognosis

**Figure 1.**
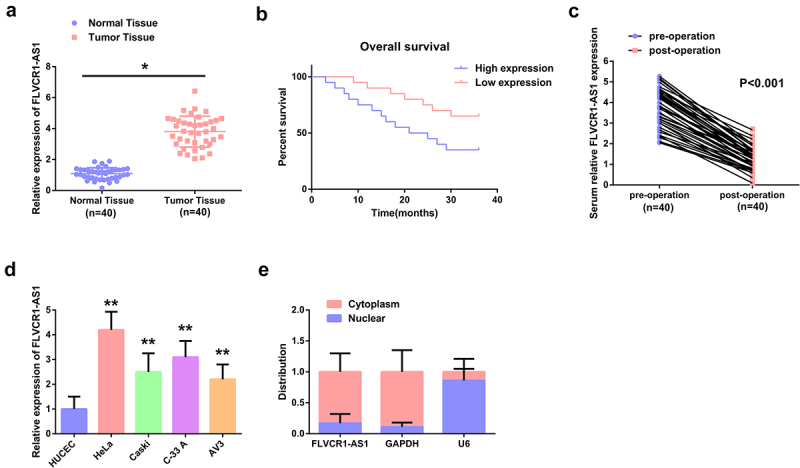
Overexpression of FLVCR1-AS1 in CC tissues and cell lines predicts unpleasing prognosis A: FLVCR1-AS1 in CC tissue; B: Kaplan-Meier analysis of the survival prognosis in CC patients; C: FLVCR1-AS1 in the serum of CC patients before and after surgery; D: FLVCR1-AS1 in CC cells; E: Subcellular localization analysis assessment of the distribution of FLVCR1-AS1 in the cytoplasm and nucleus of HeLa cells; A, C, D, RT-qPCR detection. Comparison of the data was via adopting t test, and comparison of c panel data was via adopting two-way ANOVA. * *P* < 0.05 vs. the Normal Tissue, n = 40; ** *P* < 0.05 vs. the HUCEC, N = 3.

In this research, construction of the control (para-cancerous normal tissue in CC patients) and the model (tumor tissue in CC patients) was performed. Examination of FLVCR1-AS1 in CC tissue clarified FLVCR1-AS1 was distinctively elevated in CC tissues vs. the para-cancerous normal tissues (*P* < 0.05) (Figure 1(a)). Observation of the association of FLVCR1-AS1 with patient clinical parameters in patients was performed. As manifested in Table 2, FIGO staging and lymph node metastasis were distinctively linked with FLVCR1-AS1 among the six clinicopathological characteristics, (*P* < 0.05), and other clinicopathological features were not distinctly associated with FLVCR1-AS1 (*P* > 0.05). Survival analysis (Kaplan-Meier) elucidated elevated FLVCR1-AS1 in CC tissue was distinctively linked with unpleasing overall survival (Figure 1(b)). In addition, FLVCR1-AS1 expression was inhibited in postoperative serum samples compared with preoperative serum samples (n = 40) (Figure 1(c)). These data suggested that high expression of FLVCR1-AS1 was associated with poor prognosis in patients with CC.

**Table 2.** The association of lncRNA FLVCR1-AS1 with clinicopathological characteristics in CC patients

Characteristic	Cases	LncRNA FLVCR1-AS1 expression		*P*
	n = 40	The declined (n = 20)	The elevated (n = 20)	
Age (year)				0.179
Less than 45	17	11	6	
45 or more	23	9	14	
Tumor size				0.286
Less than 4 cm	18	7	11	
4 cm or more	22	13	9	
Histology				0.313
Squamous cell carcinoma	30	17	13	
Adenocarcinoma	10	3	7	
Differentiation				0.357
Negative	25	14	11	
Positive	15	6	9	
Lymph node metastasis				0.035
No	30	17	13	
Yes	10	3	7	
FIGO stage				0.013
I, II	21	14	7	
II, IV	11	2	9	

FIGO Stage: Classification of CC

Subsequently, detection of FLVCR1-AS1 in CC and normal cervical epithelial cells was performed. The results were manifested in Figure 1(d), FLVCR1-AS1 in CC cells (HeLa, Caski, C-33 A, AV3) was declined vs. the normal cervical epithelial cells (HUCEC) (*P* < 0.05); Among the four CC cell lines, FLVCR1-AS1 was most significantly expressed in HeLa. Therefore, HeLa cells were selected for further experiments. The localization of FLVCR1-AS1 in HeLa cells was figured out. As presented in Figure 1(e), crucial distribution of FLVCR1-AS1 was in the cytoplasm of HeLa cells, manifesting that FLVCR1-AS1 exerts a crucial action in the cytoplasm of CC cells.

The above data elaborated elevated FLVCR1-AS1 in CC tissues and cell lines was linked with the poor pathological characteristics and prognosis in CC.

### 3.2. FLVCR1-AS1 promotes the malignant phenotype of HeLa cells

**Figure 2.**
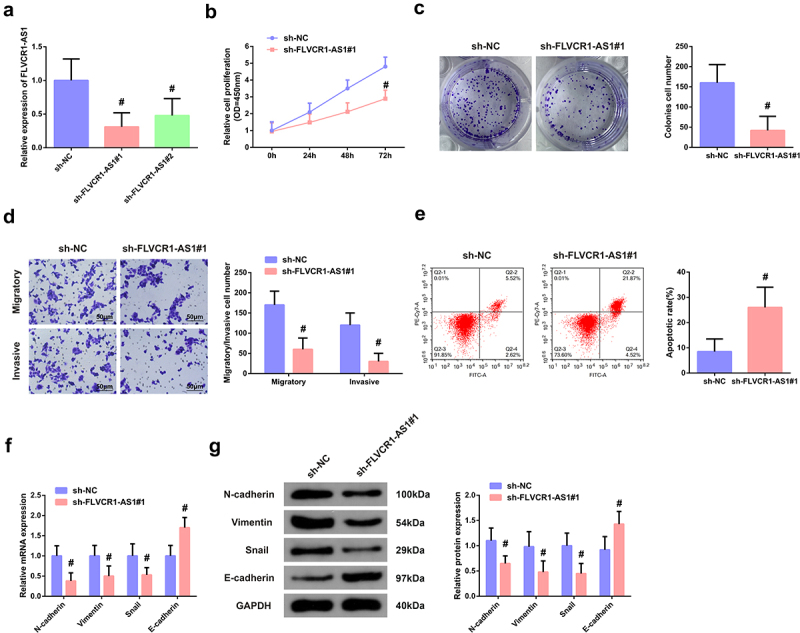
FLVCR1-AS1 promotes the malignant phenotype of HeLa cells A: RT-qPCR test of the transfection efficiency of FLVCR1-AS1 in HeLa cells; B/C: CCK-8 method and colony formation experiment detection of cell proliferation activity; D: Flow cytometry examination of cell apoptosis rate; E: Transwell test of cell migration and invasion; F/G: RT-qPCR and Western blot examination of N-cadherin, Vimentin, Snail, and E-cadherin in HeLa cells. # *P* < 0.05 vs. the sh-NC, N = 3.

Construction of the FLVCR1-AS1 knockdown cell model was performed to further explore the function of FLVCR1-AS1 in HeLa cell advancement. The transfection efficiency was affirmed, as manifested in Figure 2(a), sh- FLVCR1-AS1#1#2 was available to distinctively restrain FLVCR1-AS1, among of which sh-FLVCR1-AS1#1 was provided with the greatly distinct suppression. Consequently, adoption of sh-FLVCR1-AS1#1 was in subsequent experiments.

Subsequently, functional experiments were carried out. First, the proliferation and apoptosis of HeLa cells were detected by CCK-8 assay, colony formation experiment, and flow cytometry. As shown in Figure 2(b-d), the results clarified that FLVCR1-AS1 knockdown also inhibited the vitality of HeLa cells but promoted cell apoptosis. Transwell was used to detect migration and invasion of HeLa cells, and it came out FLVCR1-AS1 knockdown also inhibited migration and invasion of HeLa cells (Figure 2(e)). Since the EMT process contributes to the migration and invasion of cancer cells, RT-qPCR and Western blot were performed to detect markers related to the EMT process in CC. The results were shown in figure 2(f/g), clarifying that compared with sh-NC, mRNA, and protein levels of EMT-promoting markers N-cadherin, Vimentin and Snail in HeLa cells were significantly decreased after FLVCR1-AS1 knockdown (*P* < 0.05), while the expression level of E-cadherin, a marker of epithelial cells, was significantly increased (*P* < 0.05). These results suggested that downregulation of FLVCR1-AS1 could inhibit the EMT process of CC cells.

The above data suggested downregulation of FLVCR1-AS1 expression could inhibit proliferation migration, invasion, and EMT process of HeLa cells but promote their apoptosis.

### 3.3. FLVCR1-AS1 modulates miR-23a-5p in HeLa cells

**Figure 3.**
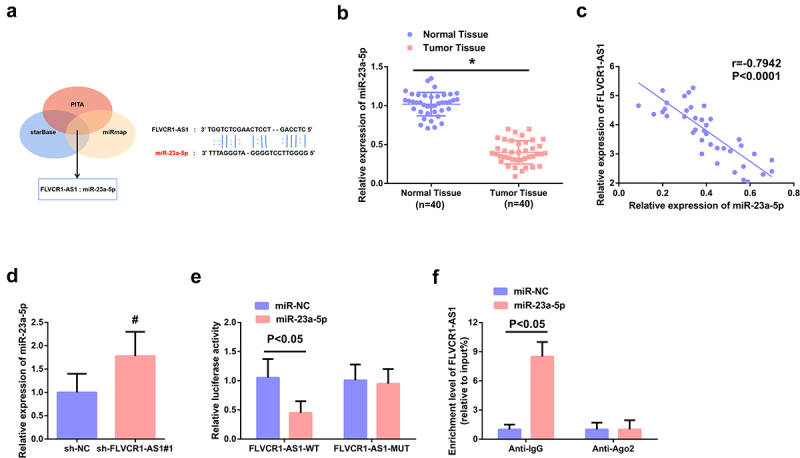
FLVCR1-AS1 modulates miR-23a-5p in HeLa cells. A: Bioinformatics website prediction of the binding site of FLVCR1-AS1 with miR-23a-5p; B: RT-qPCR detection of miR-23a-5p in CC patients (n = 40); C: Pearson correlation analysis evaluation of the association of miR-23a-5p with FLVCR1-AS1 (n = 40); D: RT-qPCR examination of miR-23a-5p after constraining FLVCR1-AS1; E/F: The luciferase activity assay and RNA-pulldown experiment assessment of the interaction of FLVCR1-AS1 with miR-23a-5p; # *P* < 0.05 vs. the sh-NC, N = 3; & *P* < 0.05 vs. the sh-NC, N = 3.

To figure out the brand-new mechanism in which FLVCR1-AS1 modulated CC, prediction of the target miRNA of FLVCR1-AS1 was via adopting bioinformatics website. MiR-23a-5p was provided with the targeted binding site of FLVCR1-AS1 (Figure 3(a)). Test of the association of FLVCR1-AS1 with miR-23a-5p was for determination of whether FLVCR1-AS1 mediated miR-23a-5p in CC. RT-qPCR results showed that the expression of miR-23a-5p was significantly down-regulated in CC patients (Figure 3(b)). Clinical correlation analysis showed that the expression of FLVCR1-AS1 was negatively correlated with the expression of miR-23a-5p (Figure 3(c)). In addition, it was found that when FlVCR1-AS1 expression was down-regulated, the expression of miR-23a-5p was increased (Figure 3(d)). The results of dual-luciferase reporter gene detection showed that miR-23a-5p overexpression could reduce the luciferase activity of FLVCR1-AS1-WT vector, while the luciferase activity of FLVCR1-AS1-MUT vector had no significant change (Figure 3(e)). RIP detection results showed that both FLVCR1-AS1 and miR-23a-5p were abundant in the RNA-induced silencing complex immunoprecipitated by Ago2 antibody but not in the RNA-induced silencing complex immunoprecipitated by IgG antibody (figure 3(f)).

The above data illuminated FLVCR1-AS1 performed as a competitive endogenous RNA (ceRNA) of miR-23a-5p.

### 3.4. Function of FLVCR1-AS1/miR-23a-5p axis in HeLa cells

**Figure 4.**
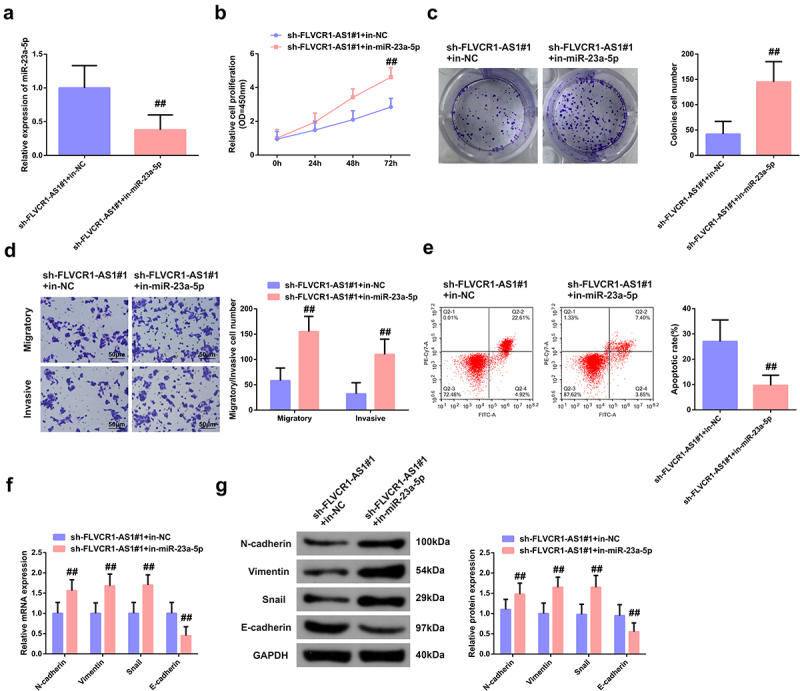
Function of FLVCR1-AS1/miR-23a-5p axis in HeLa cells. A: RT-qPCR detection of the efficiency of co-transfection of sh-LIPE-AS1#1 with in-miR-23a-5p/in-NC in HeLa cells; B/C: CCK-8 method and colony formation experiment test of cell proliferation activity; D: Flow cytometry examination of cell apoptosis rate; E: Transwell test of cell migration and invasion; F/G: RT-qPCR and Western blot detection of N-cadherin, Vimentin, Snail and E-cadherin in HeLa cells. ## *P* < 0.05 vs. the sh-LIPE-AS1#1 + in-NC, N = 3.

To further figure out the regulatory effect of FLVCR1-AS1/miR-23a-5p axis on CC cells, sh-LIPE-AS1#1 and in-miR-23a-5p or in-NC were co-transfected into HeLa cells to perform the functional rescue experiment. RT-PCR detection showed that the expression of miR-23a-5p was significantly up-regulated after transfection with sh-LIPE-AS1#1 interfering plasmid, while this reaction was significantly eliminated after co-transfection with in-miR-23a-5p (Figure 4(a)). As shown in Figure 4(b-g), results of *in vitro* functional rescue experiment clarified that miR-23a-5p inhibitor reversed the inhibitory effect of sh-LIPE-AS1#1 on proliferation, migration, invasion, and EMT process in HeLa cells, as well as the promoting effect of apoptosis. The above data illustrated that FLVCR1-AS1 could mediate the biological function of CC cells by targeting miR-23a-5p.

### 3.5. FLVCR1-AS1/miR-23a-5p targets SLC7A11

**Figure 5.**
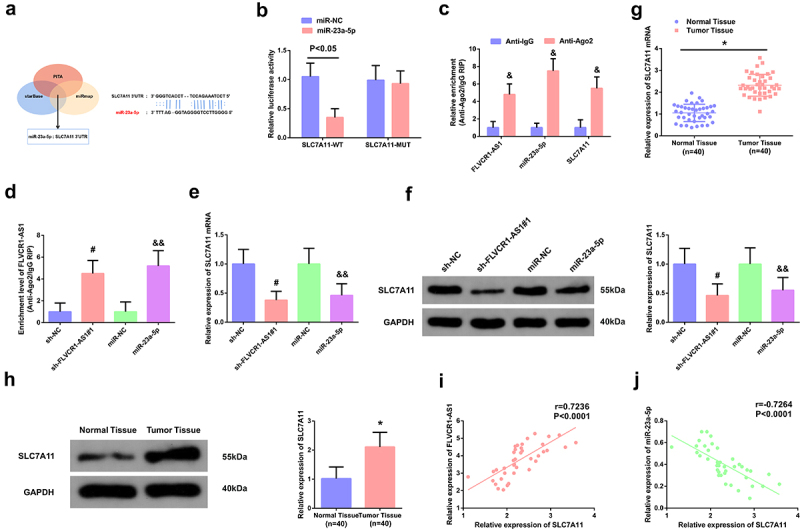
FLVCR1-AS1/miR-23a-5p targets SLC7A11. A: Bioinformatics website prediction of the binding site of SLC7A11 with miR-23a-5p; B/C/D: the luciferase activity assay and RIP experiment evaluation of the interaction of SLC7A11 with miR-23a-5p and FLVCR1-AS1; E/F: RT-qPCR and Western blot examination of SLC7A11 in HeLa cells; G/H: RT-qPCR and Western blot test of SLC7A11 in CC patients; I/J: Pearson correlation analysis assessment of the association of SLC7A11 with FLVCR1-AS1 and miR-23a-5p. & *P* < 0.05 vs. the Anti-lgG, N = 3; # *P* < 0.05 vs. the sh-NC, N = 3; && *P* < 0.05 vs. the miR-NC, N = 3; * *P* < 0.05 vs. the Normal Tissue, n = 40.

LncRNAs frequently modulated proteins via sponge to swallow miRNAs in the aspect of a ceRNA. Consequently, prediction of the downstream target of FLVCR1-AS1/miR-23a-5p was conducted, clarifying that SLC7A11 was provided with the targeted binding site of miR-23a-5p (Figure 5(a)). MiR-23a-5p nearly interacted with WT SLC7A11 mRNA 3’-untranslated region via test (Figure 5(b)). In addition, Ago2/IgG-related RIP detection showed that FLVCR1-AS1, miR-23a-5p and SLC7A11 were significantly enriched in Ago2 in HeLa cells (Figure 5(c)). In addition, down-regulation of FLVCR1-AS1 or up-regulation of miR-23a-5p significantly increased the expression of SLC7A11 enriched into Ago2 (Figure 5(d)). The experimental analysis illuminated repressive FLVCR1-AS1 or elevated miR-23a-5p was available to decline SLC7A11 (Figure 5(e-f)). Additionally, the research clarified SLC7A11 was distinctly augmented in CC tissues (Figure 5(g,h)). Clinical association analysis elaborated SLC7A11 was positively linked with FLVCR1-AS1 and reversely associated with miR-23a-5p (Figure 5(i,j)).

These data suggested that FLVCR1-AS1 acted as a competitive endogenous RNA (ceRNA) to inhibit miR-23a-5p targeting the 3’-untranslated region site of SLC7A11, thereby promoting the expression of SLC7A11.

### 3.6. MiR-23a-5p mediates the biological functions of Hela cells via SLC7A11

**Figure 6.**
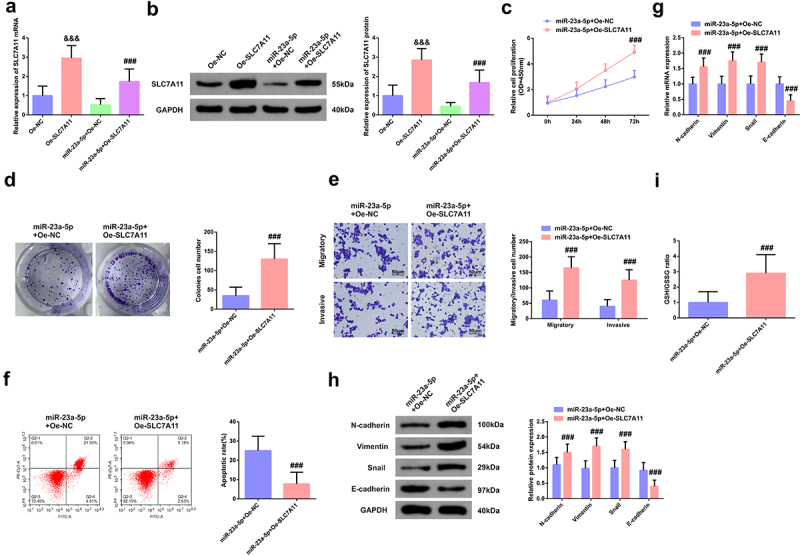
MiR-23a-5p mediates the biological functions of Hela cells via SLC7A11. A/B: Transfection efficiency of HeLa cells; C/D: CCK-8 method and colony formation experiment test of cell proliferation activity; E: Transwell examination of cell migration and invasion; F: Flow Cytometry detection of apoptosis rate; G/H: N-cadherin, Vimentin, Snail and E-cadherin in HeLa cells; I: GSH/GSSG method examination of the ratio of GSH and GSSG in HeLa cells. A, B, G, H, RT-qPCR and Western blot test. &&& *P* < 0.05 vs. the Oe-NC, N = 3; ### *P* < 0.05 vs. the miR-23a-5p + Oe-NC, N = 3.

Construction of the SLC7A11 elevation vector was conducted for further verification of the modulation of the FLVCR1-AS1/miR-23a-5p/SLC7A11 axis. After transfection of the SLC7A11 elevation vector into HeLa cells, SLC7A11 was crucially augmented (Figure 6(a,b)). Then, co-transfection of the miR-23a-5p mimic was into HeLa cells with SLC7A11 elevation vector and its negative control, and the results elaborated transfection of miR-23a-5p mimics distinctively repressed SLC7A11 in HeLa cells, while co-transfection of miR-23a-5p mimic and SLC7A11 elevation plasmid mitigated this alteration (Figure 6(a,b)).

The results elucidated elevated SLC7A11 was available to turn around augmented miR-23a-5p on the enhancement of HeLa cell viability and the silence of apoptosis (Figure 6(c-e)). Meanwhile, overexpression of SLC7A11 could also eliminate the promotion of HeLa cell migration, invasion, EMT process, and GSH/GSSG ratio by overexpression of miR-23a-5p. (figure 6(f-i)).

The above data clarified the regulatory axis of FLVCR1-AS1/miR-23a-5p/SLC7A11 axis in CC cells boosted its cell growth and advancement with EMT and was linked with cell iron death.

## 4. Discussion

CC is the crucial reason of death in patients owing to unpleasing prognosis with elevated incidence and recurrence rate [3,21]. Nevertheless, no imperative molecular characteristics were available to be adopted to predict the recurrence and survival rates in CC patients presently. The evidence has elaborated mutations and uneven modulation of lncRNA are crucial predisposing factors for cancer [22]. Additionally, researches have clarified lncRNA implicating in cell advancement with EMT in CC cells might become the latent therapeutic target for CC and be performed as the biomarker for diagnosis or prognosis [13]. Determining the biological function and modulation mechanism of lncRNA on CC was conducive to the diagnosis and cure of CC patients. Consequently, the research was to figure out the biological function and feasible mechanism of FLVCR1-AS1 in CC. Further experiments testified FLVCR1-AS1 mediated the target gene SLC7A11 via performing as a ceRNA of miR-23a-5p, thereby restraining the progression with the process of EMT of HeLa cells.

As reported, FLVCR1-AS1 is modulated in the multiple tumors like pancreatic cancer [23], Gallbladder cancer [24] and hepatocellular carcinoma [25]. Additionally, as reported, FLVCR1-AS1 is available to be performed as the prognostic or diagnostic biomarker in glioma, and its enhanced expression predicts unpleasing prognosis in glioma patients [26]. Likewise, FLVCR1-AS1 was elevated in CC tissues and cells in the research. Additionally, augmented FLVCR1-AS1 is associated with the unpleasing pathological characteristics and prognosis of CC, manifesting that FLVCR1-AS1 might be performed as the biomarker for prognosis or diagnosis of CC, which needed to be testified via testing FLVCR1-AS1 in serum of CC in follow-up researches. Latest reports have verified FLVCR1-AS1 accelerates ovarian cancer cell progression with the process of EMT [9]. Pan *et al*. also maintain FLVCR1-AS1 distinctively constraints BC cell advancement in *vitro* [17]. In this research, suppressive FLVCR1-AS1 restrained the advancement with EMT progression of CC cells (HeLa), further supporting the viewpoint of FLVCR1-AS1 as the carcinogen.

Novel evidences have elucidated lncRNA as a ceRNA mitigates the suppression of miRNA on the post-transcription of target mRNA, and then implicates in the occurrence and development of CC. For instance, lncRNA UCA1 mediates the occurrence of EMT in CC cells via targeting miR-155 [27]. The lnc-SNHG16/miR-128 axis modulates in *vitro* advancement with the process of EMT process and tumor growth in *vivo* via the WNT/β-catenin pathway [28]. Nevertheless, the fundamental mechanism of FLVCR1-AS1 in CC remains uncertain. Consequently, prediction of the targeted miRNA of FLVCR1-AS1 was conducted, and miR-23a-5p was discovered to be provided with the targeted binding site of FLVCR1-AS1. Notably, researches have elucidated miR-23a-5p is silenced in cancer and performs as the tumor suppressor gene [29]. For instance, elevated miR-23a-5p restrains cancer cell advancement in glioblastoma [30]. MiR-23a-5p is distinctly modulated in pancreatic ductal adenocarcinoma and constrains its progression [31]. In this study, miR-23a-5p also performed as the tumor suppressor gene in CC. Competitive adsorption of miR-23a-5p was via FLVCR1-AS1, and elevated miR-23a-5p restrained the malignant behavior of HeLa cells.

Iron poisoning, a kind of programmed cell death, is different from apoptosis, autophagy, and necrosis [32]. Recently, induction of cell iron death is regarded as the prospective method for the cure of cancer [16]. SLC7A11, a subunit of amino acid transport system Xc−, primarily introduces cystine into the cells during the exchange of glutamate [14]. As the crucial regulator of iron poisoning in cancer cells, SLC7A11 as the crucial regulator of iron poisoning in cancer cells is available to be modulated in the transcription, while declined SLC7A11 is able to stimulate iron poisoning [32]. Foregoing reports have elaborated SLC7A11 is nearly associated with the occurrence and advancement of tumors. For instance, lncRNA OIP5-AS1 boosts prostate cancer progression and resistance of cell iron death via miR-128-3p/SLC7A11 pathway [16]. Notably, in another report, the circEPSTI1-miR-375/409-3P/515-5p-SLC7A11 axis impacts the advancement of CC via a ceRNA mechanism, and is linked with cell iron death [33]. The research testified SLC7A11 was the downstream gene of miR-23a-5p, which targeted SLC7A11, and then influenced the progression with the progress of EMT of CC cells, and is associated with cell iron death. Additionally, the foregoing study has manifestedMiR-139-5p/SLC7A11 refrained the advancement of pancreatic cancer via the PI3K/Akt pathway [14]. MiR-139-5p/SLC7A11 restrains the progression of pancreatic cancer via PI3K/Akt pathway [14]. Consequently, a speculation that SLC7A11 might participate in the advancement of CC via targeting PI3K/Akt pathway was manifested, which needed to be further figured out in subsequent researches.

In spite of its limitations, the research discovered FLVCR1-AS1 impacted the progression of CC via modulating the miR-23a-5p/SLC7A11 axis. Nevertheless, SLC7A11 impacted the advancement of CC remains to be further figured out. Additionally, the number of patient samples in this research was not enough and needed to be augmented to testify the diagnosis and cure of FLVCR1-AS1. Multi-center trials and animal experiments should be performed to illuminate the action of FLVCR1-AS1 in CC later.

## 5. Conclusion

This study shows that FLVCR1-AS1 is one of the important regulatory factor of CC progress. FLVCR1-AS1 silencing can inhibit the proliferation, migration, invasion, and EMT process of CC cells, but promote cell apoptosis. FLVCR1-AS1 affects the progression of CC through a regulatory network composed of FLVCR1-AS1, miR-23a-5p, and SLC7A11. The study provides a promising target for the treatment of CC.

## References

[1] Yuan D, Jia C-D, Yan M-Y, et al. Circular RNA hsa_circ_0000730 restrains cell proliferation, migration, and invasion in cervical cancer through miR −942-5p/ PTEN axis. Kaohsiung J Med Sci. 2021;37(11):964–972. 34562344 10.1002/kjm2.12443 PMC11896307

[2] Huang X, Shi H, Shi X, et al. LncRNA FBXL19-AS1 promotes proliferation and metastasis of cervical cancer through upregulating COL1A1 as a sponge of miR-193a-5p. J Biol Res. 2021;28(1):20. 10.1186/s40709-021-00151-8 PMC8365943 34399848

[3] Zhai Y, Liu Y, Wang Z, et al. Long non-coding RNA LINC00313 accelerates cervical carcinoma progression by miR-4677-3p/CDK6 axis. Onco Targets Ther. 2021;14:2213–2226. 33824592 10.2147/OTT.S265007 PMC8018412

[4] Liu L, Wang M, Li X, et al. An overview of novel agents for cervical cancer treatment by inducing apoptosis: emerging drugs ongoing clinical trials and preclinical studies. Front Med (Lausanne). 2021;8:682366. 34395473 10.3389/fmed.2021.682366 PMC8355560

[5] Lee Y, Choi MC, Park JY, et al. Major clinical research advances in gynecologic cancer in 2020. J Gynecol Oncol. 2021;32(4):e53. 34085794 10.3802/jgo.2021.32.e53 PMC8192228

[6] Liu D, Huang K, Wang T, et al. NR2F2-AS1 accelerates cell proliferation through regulating miR-4429/MBD1 axis in cervical cancer. Biosci Rep. 2020;40(6). 10.1042/BSR20194282 PMC7295628 32469064

[7] Zhang J, Jiang P, Wang S, et al. LncRNA LIPE-AS1 predicts poor survival of cervical cancer and promotes its proliferation and migration via modulating miR-195-5p/MAPK pathway. Front Oncol. 2021;11:639980. 33898314 10.3389/fonc.2021.639980 PMC8062982

[8] Jiang S, Kong P, Liu X, et al. LncRNA FLVCR1-AS1 accelerates osteosarcoma cells to proliferate, migrate and invade via activating wnt/β-catenin pathway. J BUON. 2020;25(4):2078–2085. 33099956

[9] Yan H, Li H, Silva MA, et al. LncRNA FLVCR1-AS1 mediates miR-513/YAP1 signaling to promote cell progression, migration, invasion and EMT process in ovarian cancer. J Exp Clin Cancer Res. 2019;38(1):356. 31412903 10.1186/s13046-019-1356-z PMC6694549

[10] Shi Y, Zhang Q, Xie M, et al. Aberrant methylation-mediated decrease of lncRNA HNF1A-AS1 contributes to malignant progression of laryngeal squamous cell carcinoma via EMT. Oncol Rep. 2020;44(6):2503–2516. 33125127 10.3892/or.2020.7823 PMC7640355

[11] Wang J, Liu Y, Cai H, et al. Long coding RNA CCAT2 enhances the proliferation and epithelial-mesenchymal transition of cervical carcinoma cells via the microRNA-493-5p/CREB1 axis. Bioengineered. 2021;12(1):6264–6274. 34499007 10.1080/21655979.2021.1969834 PMC8806934

[12] Liu H, Zhang L, Ding X, et al. LINC00861 inhibits the progression of cervical cancer cells by functioning as a ceRNA for miR-513b-5p and regulating the PTEN/AKT/mTOR signaling pathway. Mol Med Rep. 2021;23(1). 10.3892/mmr.2020.11662 PMC7673320 33179755

[13] Sun Z, Hu J, Hu K, et al. [Role of long noncoding RNA SNHG3 in regulating proliferation, migration and invasion of cervical cancer SiHa cells]. Nan Fang Yi Ke Da Xue Xue Bao. 2021;41(6):931–936. 34238747 10.12122/j.issn.1673-4254.2021.06.17 PMC8267985

[14] Zhu J, De Mello RA, Yan Q-L, et al. MiR-139-5p/SLC7A11 inhibits the proliferation, invasion and metastasis of pancreatic carcinoma via PI3K/Akt signaling pathway. Biochim Biophys Acta Mol Basis Dis. 2020;1866(6):165747. 32109492 10.1016/j.bbadis.2020.165747

[15] Wu Q, Lu S, Zhang L, et al. LncRNA HOXA-AS2 activates the notch pathway to promote cervical cancer cell proliferation and migration. Reprod Sci. 2021;28(10):3000–3009. 34076871 10.1007/s43032-021-00626-y

[16] Zhang Y, Guo S, Wang S, et al. LncRNA OIP5-AS1 inhibits ferroptosis in prostate cancer with long-term cadmium exposure through miR-128-3p/SLC7A11 signaling. Ecotoxicol Environ Saf. 2021;220:112376. 34051661 10.1016/j.ecoenv.2021.112376

[17] Pan Z, Ding J, Yang Z, et al. LncRNA FLVCR1-AS1 promotes proliferation, migration and activates Wnt/β-catenin pathway through miR-381-3p/CTNNB1 axis in breast cancer. Cancer Cell Int. 2020;20(1):214. 32518523 10.1186/s12935-020-01247-2 PMC7275497

[18] Zhang X, Wang Y, Zhao A, et al. Long Non-Coding RNA LINC00511 accelerates proliferation and invasion in cervical cancer through targeting miR-324-5p/DRAM1 axis. Onco Targets Ther. 2020;13:10245–10256. 33116605 10.2147/OTT.S255067 PMC7567551

[19] Ogawa K, Noda A, Ueda J, et al. Forced expression of miR-143 and −145 in cardiomyocytes induces cardiomyopathy with a reductive redox shift.[J]. Cell Mol Biol Lett. 2020;25:40. 32855642 10.1186/s11658-020-00232-x PMC7444248

[20] Wang H, Ma J, Zhan X. Circular RNA Circ_0067934 attenuates ferroptosis of thyroid cancer cells by miR-545-3p/SLC7A11 signaling. Front Endocrinol (Lausanne). 2021;12:670031. 34290668 10.3389/fendo.2021.670031 PMC8287831

[21] Li M, Tian X, Guo H, et al. A novel lncRNA-mRNA-miRNA signature predicts recurrence and disease-free survival in cervical cancer. Braz J Med Biol Res = Rev Bras Pesqui Med Biol. 2021;54(11):e11592. 10.1590/1414-431X2021e11592 PMC8457683 34550275

[22] Yuan X, Zhao Q, Zhang Y, et al. The role and mechanism of HLA complex group 11 in cancer. Biomed Pharmacothe. 2021;143:112210. 10.1016/j.biopha.2021.112210 34563948

[23] Lin J, Zhai S, Zou S, et al. Positive feedback between lncRNA FLVCR1-AS1 and KLF10 may inhibit pancreatic cancer progression via the PTEN/AKT pathway. J Exp Clin Cancer Res. 2021;40(1):316. 34635142 10.1186/s13046-021-02097-0 PMC8507233

[24] Bao W, Cao F, Ni S, et al. lncRNA FLVCR1-AS1 regulates cell proliferation, migration and invasion by sponging miR-485-5p in human cholangiocarcinoma. Oncol Lett. 2019;18(3):2240–2247. 31404302 10.3892/ol.2019.10577 PMC6676717

[25] Zhang K, Zhao Z, Yu J, et al. LncRNA FLVCR1-AS1 acts as miR-513c sponge to modulate cancer cell proliferation, migration, and invasion in hepatocellular carcinoma. J Cell Biochem. 2018;119(7):6045–6056. 29574975 10.1002/jcb.26802

[26] Yan Z, Zhang W, Xiong Y, et al. Long noncoding RNA FLVCR1-AS1 aggravates biological behaviors of glioma cells via targeting miR-4731-5p/E2F2 axis. Biochem Biophys Res Commun. 2020;521(3):716–720. 31699367 10.1016/j.bbrc.2019.10.106

[27] Yang T, Wang L, Zhang Y, et al. LncRNA UCA1 regulates cervical cancer survival and EMT occurrence by targeting miR-155. Eur Rev Med Pharmacol Sci. 2020;24(19):9869–9879. 33090390 10.26355/eurrev_202010_23197

[28] Wu W, Guo L, Liang Z, et al. Lnc-SNHG16/miR-128 axis modulates malignant phenotype through WNT/β-catenin pathway in cervical cancer cells. J Cancer. 2020;11(8):2201–2212. 32127947 10.7150/jca.40319 PMC7052928

[29] Bao X, Ma L, He C. MicroRNA-23a-5p regulates cell proliferation, migration and inflammation of TNF-α-stimulated human fibroblast-like MH7A synoviocytes by targeting TLR4 in rheumatoid arthritis. Exp Ther Med. 2021;21(5):479. 33767774 10.3892/etm.2021.9910 PMC7976437

[30] Gao X, Cao Y, Li J, et al. LncRNA TPT1-AS1 sponges miR-23a-5p in glioblastoma to promote cancer cell proliferation. Cancer Biother Radiopharm. 2020;36(7):549–555. 32783743 10.1089/cbr.2019.3484

[31] Huang W, Huang Y, Gu J, et al. miR-23a-5p inhibits cell proliferation and invasion in pancreatic ductal adenocarcinoma by suppressing ECM1 expression. Am J Transl Res. 2019;11(5):2983–2994. 31217868 PMC6556669

[32] Lyu N, Zeng Y, Kong Y, et al. Ferroptosis is involved in the progression of hepatocellular carcinoma through the circ0097009/miR-1261/SLC7A11 axis. Ann Transl Med. 2021;9(8):675. 33987373 10.21037/atm-21-997 PMC8106082

[33] Wu P, Li C, Ye DM, et al. Circular RNA circEPSTI1 accelerates cervical cancer progression via miR-375/409-3P/515-5p-SLC7A11 axis. Aging (Albany NY). 2021;13(3):4663–4673. 33534779 10.18632/aging.202518 PMC7906137

